# SCNBase: a genomics portal for the soybean cyst nematode (*Heterodera glycines*)

**DOI:** 10.1093/database/baz111

**Published:** 2019-11-13

**Authors:** Rick Masonbrink, Tom R Maier, Arun S Seetharam, Parijat S Juvale, Levi Baber, Thomas J Baum, Andrew J Severin

**Affiliations:** 1 Genome Informatics Facility, Iowa State University, Osborne Dr, Ames, IA 50011, USA; 2 Department of Plant Pathology and Microbiology, Iowa State University, Pammel Dr, Ames, IA 50011, USA; 3 Research IT, Iowa State University, Osborne Dr, Ames, IA 50011, USA

## Abstract

Soybean is an important worldwide crop, and farmers continue to experience significant yield loss due to the soybean cyst nematode (SCN), *Heterodera glycines*. This soil-borne roundworm parasite is rated the most important pathogen problem in soybean production. The infective nematodes enter into complex interactions with their host plant by inducing the development of specialized plant feeding cells that provide the parasites with nourishment. Addressing the SCN problem will require the development of genomic resources and a global collaboration of scientists to analyze and use these resources. SCNBase.org was designed as a collaborative hub for the SCN genome. All data and analyses are downloadable and can be analyzed with three integrated genomic tools: JBrowse, Feature Search and BLAST. At the time of this writing, a number of genomic and transcriptomic data sets are already available, with 43 JBrowse tracks and 21 category pages describing SCN genomic analyses on gene predictions, transcriptome and read alignments, effector-like genes, expansion and contraction of genomic repeats, orthology and synteny with related nematode species, Single Nucleotide Polymorphism (SNPs) from 15 SCN populations and novel splice sites. Standard functional gene annotations were supplemented with orthologous gene annotations using a comparison to nine related plant-parasitic nematodes, thereby enabling functional annotations for 85% of genes. These annotations led to a greater grasp on the SCN effectorome, which include over 3324 putative effector genes. By designing SCNBase as a hub, future research findings and genomic resources can easily be uploaded and made available for use by others with minimal needs for further curation. By providing these resources to nematode research community, scientists will be empowered to develop novel, more effective SCN management tools.

## Introduction

Worldwide, soybean serves as a major nutritive component of human and livestock diets, providing high-quality protein and oil. Demand for soybean is likely to continue to rise with increasing populations and recent shifts in dietary trends. It is essential to maximize soybean production to support global food security. Yet, many biotic and abiotic stresses continually impact soybean yield worldwide. The most prominent of these is caused by a soil-borne parasitic worm *Heterodera glycines*, the soybean cyst nematode (SCN). SCN is the most economically damaging soybean pest, causing upwards of $1.2 billion dollars of yield loss each year in the USA (1). SCN management has traditionally relied upon a combination of crop rotation, the use of SCN-resistant soybean varieties and nematicides. However, the current practice is losing its potency. SCN-resistant soybean varieties are increasingly becoming vulnerable to SCN infections, as genetic variability, numerous progeny and a relatively short life cycle have enabled the evolution of SCN populations to overcome resistant soybean varieties. The narrow genetic basis of soybean’s SCN resistance and the overuse of these varieties have exacerbated the problem by stimulating the selection of virulent SCN populations throughout many soybean production areas (2–8). Thus, there is an immediate need to identify and develop novel management approaches to combat this pest.

SCN has evolved complex and fine-tuned molecular interactions with the soybean plant that culminate into the long-term suppression of effective defense responses and the development of a feeding structure in the soybean root, the syncytium. The SCN lifecycle begins with second-stage juveniles (J2) hatching from eggs in the soil and migrating toward host roots. J2s destructively enter and migrate through the root to find a preferred feeding location, usually in the vicinity of the vascular tissue. Once found, the nematode becomes sedentary and elicits host cell reprogramming to create cells that sequester nutrients from the vascular system, a process likely initiated by delivering secreted proteins (effectors) into the plant tissue using its hollow mouth spear, the stylet (1–8). Many long-standing questions in the biology of this parasite remain. It is particularly important to identify and functionally characterize all SCN effectors and to understand their variability among nematode populations with differing levels of virulence. Such analyses likely will reveal the genetic components responsible for SCN’s ability to overcome defense mechanisms in previously resistant soybean cultivars. In pursuit of these questions, a number of diverse secreted effector proteins have been identified that enable SCN to (6, 9–16) mimic host regulatory peptides (17, 18), to affect hormone transport and signaling (19, 20), to modify polyamine metabolism (21), to suppress the host immune system (6, 22–24), remodel host chromatin (25), to modify cell walls (26) and to utilize the host proteolytic pathway (27). Thus, understanding the genetic basis for these SCN phenotypes will better inform soybean researchers and breeders on how to develop soybean cultivars with lasting SCN resistance.

The assembly of an SCN genome sequence has enabled the application of a number of new omics technologies that may prove insightful to SCN research. While WormBase (28) and ParaSite (29) websites are great resources for all currently sequenced nematode genomes, gene annotations and other general omics data, species-specific databases are needed to mitigate the deluge of data detailing the intricacies of the host–parasite relationships. To fully exploit such a wealth of data, we created SCNBase, a globally accessible web portal to centralize SCN omics. We incorporated all currently available public data into SCNBase, including the supporting data from the SCN genome and transcriptome (30, 31). Many pre-existing analyses are available, including gene predictions, promoters, raw data alignments, genomic SNPs from 15 populations, synteny and ortholog families for related species, mitochondrial genome annotations, functional protein domains and effector predictions and alignments.

We improved existing genomic resources by including new analyses to improve current genome annotations. The functional annotation of genes in draft genomes can be extremely low compared to model species (32). However, we addressed this issue in the SCN genome, as exceptional detail has been added to gene annotations that uniquely incorporates orthologous annotations from nine related plant-parasitic nematodes. Repeat expansions and contractions were further surveyed using RepeatExplorer, adding depth to repeat annotations that may have been removed during the purging of heterozygous scaffolds during assembly. SCNBase will continue to evolve by facilitating community involvement and curation through an interface that is easy to use, view and search. Our goal with SCNBase is to bridge the gap between SCN and soybean researchers by increasing collaboration to better understand the extant diversity and adaptive mechanisms of SCN.

## Materials and Methods

### Data sets

Genomes, proteins and GFF files were downloaded from WormBase (29) for *Globodera rostochiensis* (33), *Globodera pallida* (34), *Meloidogyne hapla* (35), *Meloidogyne incognita* (36), *Meloidogyne floridensis* (37), *Meloidogyne javanica* (37), *Ditylenchus destructor* (38) and *Bursaphelenchus xylophilus* (39). The *Globodera ellingtonae* (40) genome was downloaded from NCBI, and gene annotations were created in the *H. glycines* genome (31).

### Ortholog-based annotation

Proteins were renamed using Maker scripts (41). Orthologous proteins were identified using Orthofinder (42) under default conditions using primary isoforms only. Once ortholog families were established, all annotations from each protein were extracted using custom Bash scripts. The custom ortholog-based annotations were then counted using matches to the column nine annotations of each GFF file and then sorted by predominance. Scripts are available at https://github.com/remkv6/SCNBase and SCNBase.org.

### RepeatExplorer

*H. glycines* strain TN20 150 bp PE DNA-seq reads were downloaded from NCBI (SRR1800548) trimmed with Trimmomatic (43), converted to fasta with the FASTX-Toolkit (44), sorted and interlaced using Seqtk (45). After trimming adapters and low-quality sequence, 200 000 read pairs were retained that maintained the maximum length (150 bp). Classified repeats were also obtained using RepeatModeler (46) on the *H. glycines* genome (31). These reads were submitted to Repeatexplorer.org webserver (47) with paired = true, rename sequences = true, min overlap for clustering = 55, cluster size threshold for detailed analysis = 0.002, RepeatMasker database = Metazoa, custom repeat database = true, sequence filtering = false and minimal overlap for assembly = 50. The fasta output from RepeatExplorer was aligned to the genome using Gmap (default parameters) (48).

### Database development

SCNBase uses GMOD (49), Tripal (50) and Drupal’s luggage extension to manage the genomics data and empower efficient web development by multiple users. Tripal is based on a community-derived database schema named Chado (51, 52) and employs the use of controlled vocabularies such as Sequence Ontology (53), Gene Ontology (54) and others to ensure standardization of data storage.

## Results and Discussion

### Homepage

**Figure 1:**
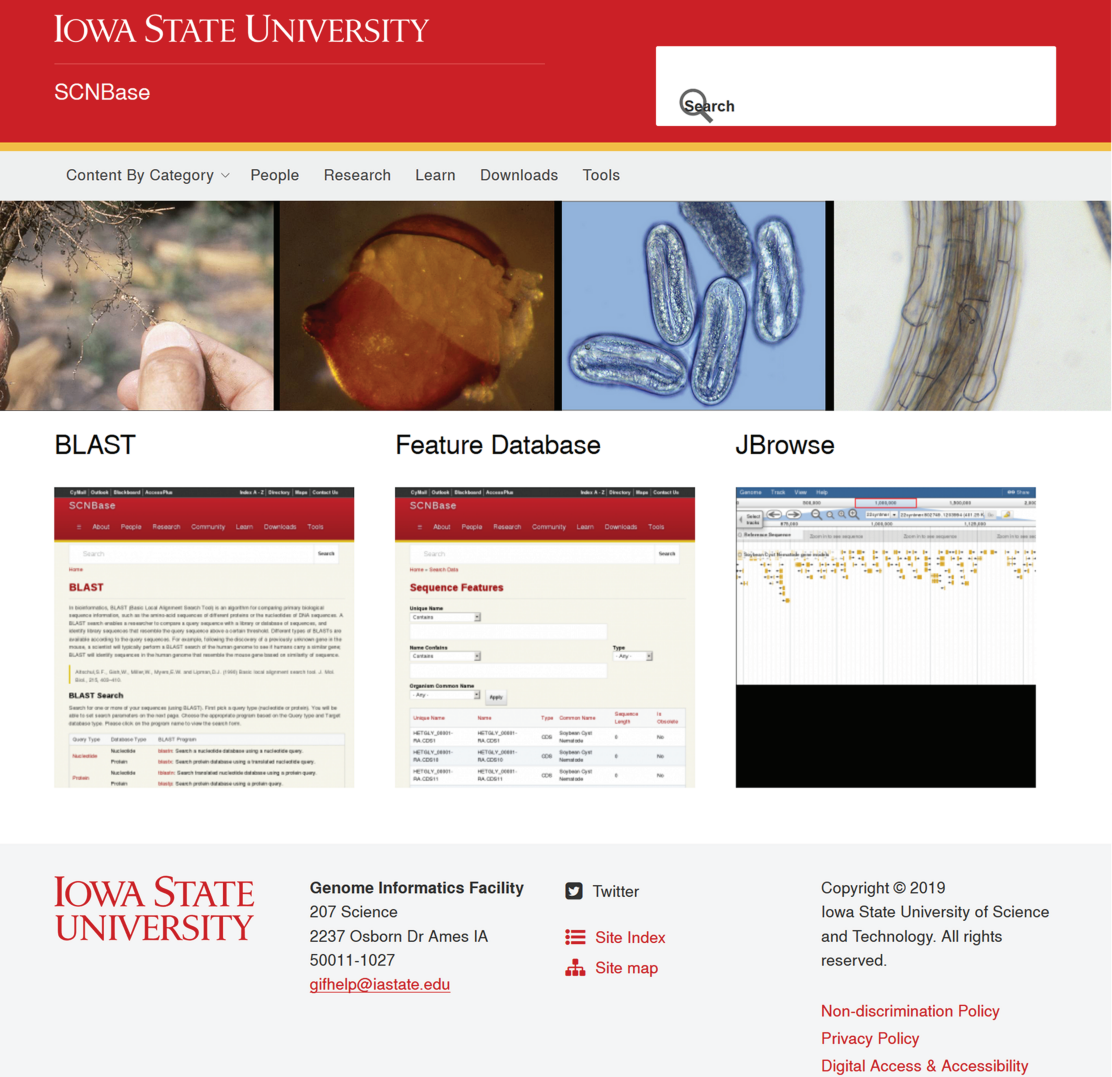
SCNBase.org homepage. The homepage provides a quick access to the most frequently used functions, bioinformatics tools and search functions. Content pages are found in the ‘Content by Category’ links.

Because a functional, user-friendly and attractive homepage is likely to garner more interest from the end-users, we created a simple interface (Figure 1). The menu at the top of the homepage includes six choices: ‘Content By Category’, ‘People’, ‘Research’, ‘Learn’, ‘Downloads’ and ‘Tools’. The ‘Content By Category’ menu provides quick links to categories of data on the website, including ‘Comparative Biology’, ‘Effector Biology’, ‘Omics’, ‘Research’, ‘Statistics’ and ‘Tools’. The ‘People’ choice describes the researchers currently collaborating in maintenance and database curation. The ‘Research’ link provides introductory information on current research in the SCN community. ‘Learn’ provides a link to frequently asked questions, as well as the possibility to ask a question. The ‘Downloads’ option directs the user to a list of downloadable genomics content, while ‘Tools’ links the user to all available genomics tools.

The information embedded within the ‘Content By Category’ menu details different genomic analyses and provides supporting information for alignment tracks. ‘Comparative Biology’ leads the user to synteny and orthology data and experiments, while ‘Effector Biology’ provides data that are relevant to effector/parasite research. The ‘Omics’ category contains all general genomics analyses, including repeats, genome contamination, mitochondrial sequences, genomic structures, gene subsets and novel splicing. ‘Research’ leads the user to active and ongoing SCN research projects. ‘Statistics’ encompasses numerical data on the genome assembly and annotation, and ‘Tools’ leads the user to available genomics tools.

The homepage is designed to draw the eye by displaying different SCN life stages, ‘parasitized soybean roots’, ‘egg-filled cyst’, ‘eggs’ and ‘infecting J2’, but also supplies the user with immediate access to a number of tools. The top right of the page hosts a search bar integrated with all site content, including research projects, people, tools, frequently asked questions, file metadata and functional gene annotations. The most frequently used tools are present at the lower end of the homepage: JBrowse, BLAST and Feature Search. The JBrowse genome browser comes with a comprehensive selection of informative and downloadable tracks (Table **1**). BLAST is available to search the genome, CoDing Sequence (CDS) and predicted proteome using all four BLAST algorithms: BLASTn, BLASTx, BLASTp and tBLASTn. The Feature Search tool provides an avenue to investigate the gene features and functions of SCN gene annotations, including sequences, functional cross-references, orthologous gene annotations and gene ontology. Altogether, with the simplicity of the layout, the far-reaching functionality of the search tool, and the in-depth list of tutorials, we hope to inspire users to use SCNBase for their research.

### Genomic resources available on SCNbase

The recently published *H. glycines* TN10 genome (31) provides a foundation for 43 JBrowse tracks and 21 category pages detailing genomic analyses/resources of gene prediction, genomic structure, transcripts, genomic and transcriptional read alignments, effector predictions, repeats, orthologs, synteny, SNPs and novel splicing. Some of the most powerful public expression data sets are present with 230 million RNA-seq reads enabling the scrutinization of gene expression changes across pre-parasitic and parasitic nematodes on resistant and susceptible soybean cultivars (30). A long-read transcript (IsoSeq) data set improves gene models and enables the confident assessment of splicing isoforms across multiple life stages in virulent and avirulent populations (31).

**Table 1:** The data in SCNBase.org allocated by data type, name, JBrowse alignment and category page content. A total of 43 JBrowse alignments and 21 category pages are available

**Data Type**	**Data Name**	**Alignment**	**Page**
Gene	High Confidence Horizontal Gene Transfers	+	
Gene	Alignments of genes producing secreted proteins	+	+
Gene	Genes with promoters containing DOG-box motifs	+	+
Gene	Alignment of 80 published known effector genes	+	+
Gene	Mitochondrial Genes	+	+
Gene	Annotated Gene Models Heterodera Glycines (updated v2)	+	
Gene	Annotated Gene Models Heterodera Glycines (v2) IPRSCAN	+	
Genes	Soybean Cyst Nematode (v2) gene models	+	
Genome Structure	Tandem Duplications	+	+
Genomic-seq	NCBI Nucleotide Database Entries	+	
Genomic-seq	Falcon Assembled Pacbio RS II subreads (pReads)	+	
Genomic-seq	Circular Consensus Pacbio RS II subreads (CCS Reads)	+	
Genomic-seq	Camtech Raw Pacbio RS II subreads	+	
Iso-seq	W82_PA3_Eggs Isoseq reads	+	
Iso-seq	W82_PA3_J2/J3 Isoseq reads	+	
Iso-seq	W82_PA3_J4/Adult Isoseq reads	+	
Iso-seq	W82_TN19_Eggs Isoseq reads	+	
Iso-seq	W82_TN19_J4/Adult Isoseq reads	+	
Iso-seq	W82_TN19_J2/J3 Isoseq reads	+	
Motif	80 Effector MEME->FIMO motifs	+	+
Orthologs	Globodera pallida Opscan orthologs with H. glycines		+
Orthologs	Globodera rostochiensis Opscan orthologs with H. glycines		+
Orthologs	Globodera ellingtonae Opscan orthologs with H. glycines		+
Orthologs	Meloidogyne hapla Opscan orthologs with H. glycines		+
Orthologs	Meloidogyne incognita Opscan orthologs with H. glycines		+
Orthologs	Bursephelenchus xylophilus Opscan orthologs with H. glycines		+
Repeat	RepeatModeler Repeats	+	+
Repeat	LTR Retroelements	+	
Repeat	DNA Transposons	+	
Repeat	Helitrons	+	
Repeat	Repeat Explorer of TN20	+	+
RNA-seq	Parasitic J2 (PA3) Forrest (Resistant Soybean) Biorep 2	+	
RNA-seq	Parasitic J2 (PA3) EXF63 (Susceptible soybean cultivar) Biorep 1	+	
RNA-seq	Pre-parasitic J2 (PA3) Biorep 1	+	
RNA-seq	Parasitic J2 (PA3) Forrest (Resistant Soybean) Biorep 1	+	
RNA-seq	Parasitic J2 (PA3) EXF63 (Susceptible soybean cultivar) Biorep 2	+	
RNA-seq	Pre-parasitic J2 (PA3) Biorep 2	+	
RNA-seq	SCN Soybean Root Exposure 3h-Control T01	+	
RNA-seq	SCN Soybean Root Exposure 3h-Control T02	+	
RNA-seq	SCN Soybean Root Exposure 3h-Control T11	+	
RNA-seq	SCN Soybean Root Exposure 3h-Attraction T03	+	
RNA-seq	SCN Soybean Root Exposure 3h-Attraction T04	+	
RNA-seq	SCN Soybean Root Exposure 3h-Attraction T09	+	
RNA-seq	SCN Soybean Root Exposure 24h-Control of T05	+	
RNA-seq	SCN Soybean Root Exposure 24h-Control of T06	+	
RNA-seq	SCN Soybean Root Exposure 24h-Control of T10	+	
RNA-seq	SCN Soybean Root Exposure 24h-Attraction T07	+	
RNA-seq	SCN Soybean Root Exposure 24h-Attraction T08	+	
RNA-seq	SCN Soybean Root Exposure 24h-Attraction T12	+	
SNP	SNPs across 15 populations of H.glycines	+	
Splicing	Novel splice junctions		+
Synteny	Globodera rostochiensis Synteny with H.glycines	+	+
Synteny	Meloidogyne Incognita Syntenic Regions	+	+
Synteny	Globodera ellingtonae Synteny with H.glycines	+	+
Synteny	Meloidogyne hapla Synteny with H.glycines	+	+
Synteny	Globodera pallida Synteny with H.glycines	+	+
Synteny	Meloidogyne and Globodera Synteny with H.glycines	+	+
Synteny	H.glycines genome assembly (ABLA00000000.1) synteny	+	
Transcript	PA3 Transcriptome on susceptible and resistant soybean	+	
Transcript	Consensus Isoseq Transcriptome from PA3 and TN19	+	
Transcript	NCBI EST Database Entries	+	
Transcript	Soybean Cyst Nematode (v2) transcript models	+	

### Expanding genomic data sets

**Figure 2:**
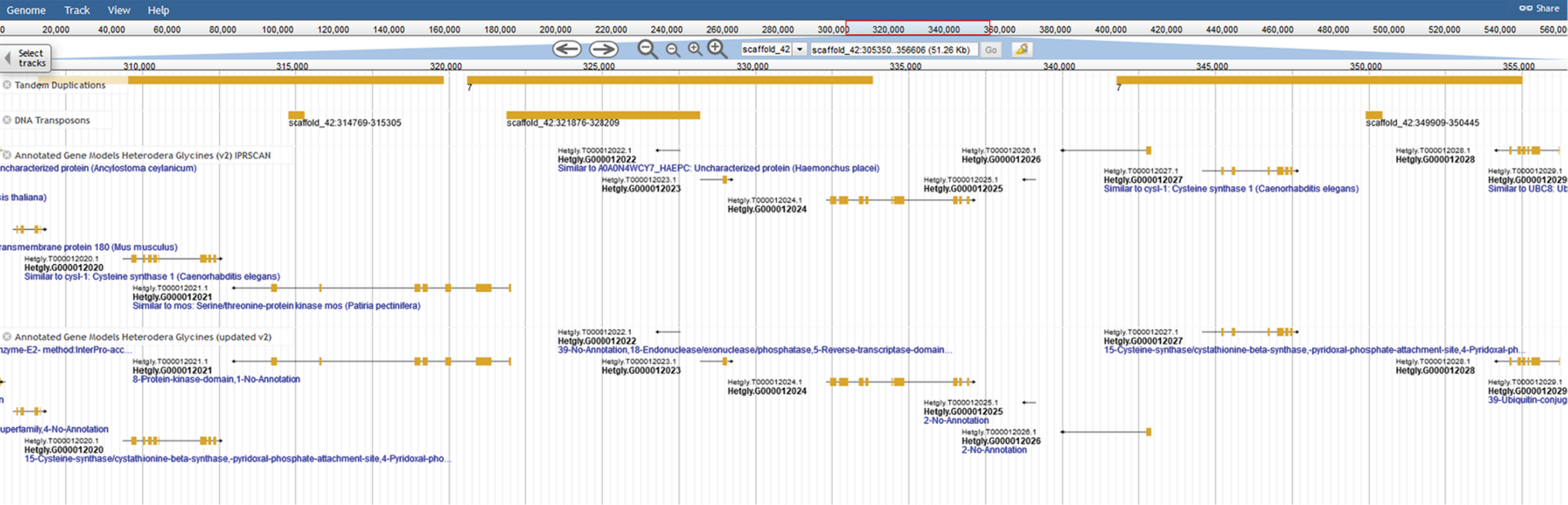
Colocalization of transposable elements, tandem duplications and genes may hint to how effector genes diversify. JBrowse annotations are in the following order: tandem duplications, DNA transposons, interpro-annotated genes and ortholog-annotated genes. Ortholog names represent the number of orthologous annotations among nine related plant-parasitic nematodes in the Tylenchia subclass.

The diversity within the phylum Nematoda, and speed with which nematodes evolve, results in large numbers of novel and unannotated genes. To fully exploit these resources and identify genes involved in parasitism, we created a robust gene annotation that leverages numerous databases and related species. Interproscan identified ontological terms and domains associated with 22 718 genes/proteins. Functional information was also obtained for 20 305 genes using orthology-based annotations to 9 sister species. Orthologous families were assigned with Orthofinder, and gene families were assigned a count of every unique gene function (Figure 2). This analysis adds depth to the annotations by capitalizing on the unique functional annotations of nine related Tylenchia species (*B. xylophilus*, *D. destructor*, *M. hapla*, *M. incognita*, *M. arenaria, M. javanica*, *M. floridensis*, *G. pallida* and *G. rostochiensis*), altogether providing functional annotations for 85% of genes (25 295/29769). Both transposable element genes (1853) and genes with effector-like functional annotations (2824) were identified as well. However, the increase in these genes is consistent with a gene prediction on an unmasked genome and consistent with effectors evolving from gene families attributed to a different function in most organisms (7).

**Figure 3:**
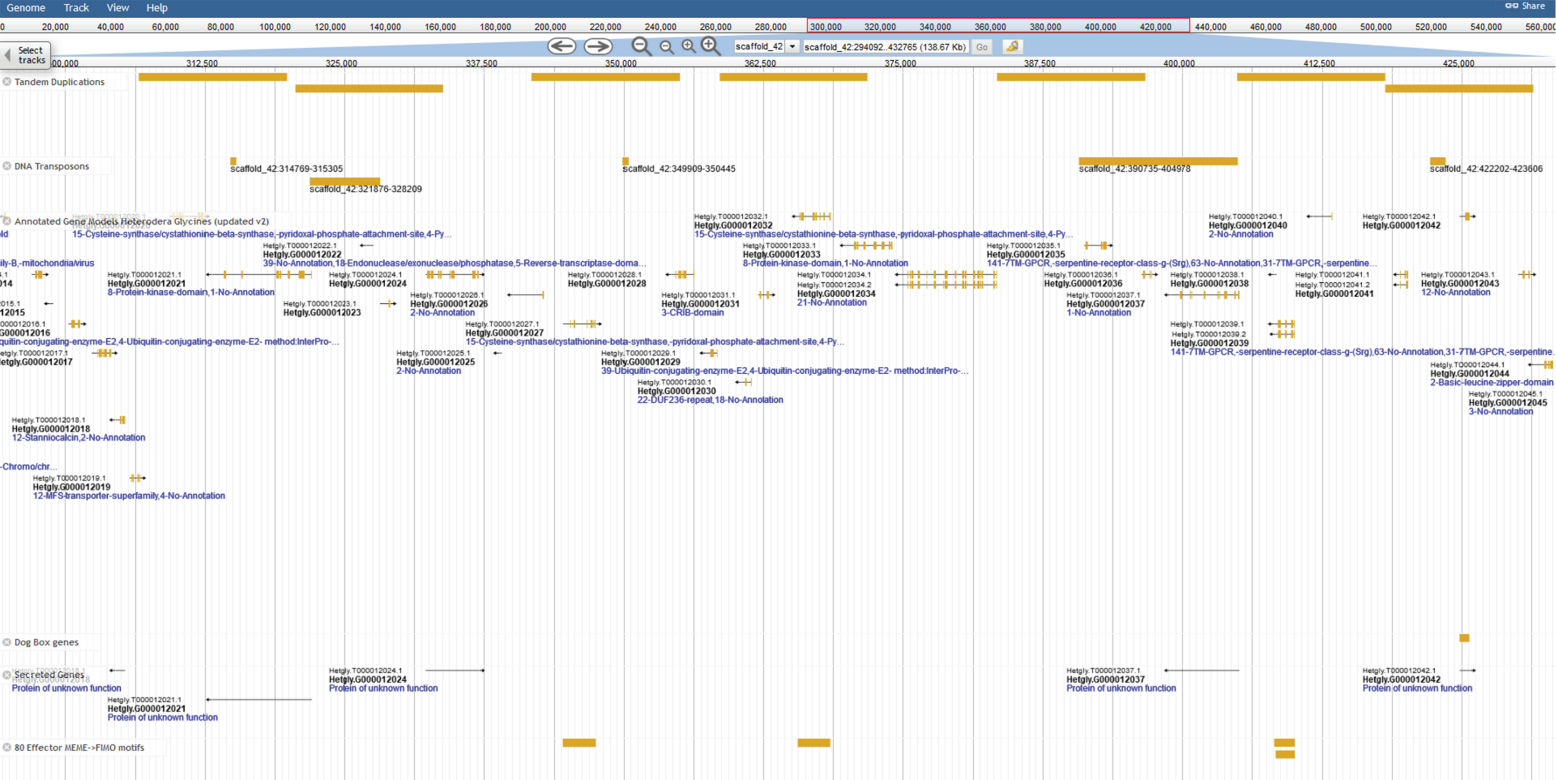
Duplication of effector genes through transposition/tandem duplication. Three types of effectors appear duplicated by tandem duplications, a DOG-box gene, multiple secreted genes and genes containing motifs derived from 80 previously published SCN effector sequences. JBrowse annotations are in the following order: tandem duplications, DNA transposons, ortholog-annotated genes, DOG-box genes, secreted genes and effector MEME motifs. Ortholog names represent the number of orthologous annotations among nine related plant-parasitic nematodes in the Tylenchia subclass.

Coincidentally, transposable elements are implicated in the duplication of effector genes in the genome (31); thus, extensive repetitive predictions were pursued. Repeats were identified with both the TN10 genome (Repeat Modeler) and from strain TN20 raw DNAseq reads (RepeatExplorer). Transposon models were created for Long Terminal Repeat LTR retrotransposons, DNA transposons and helitrons to distinguish transposon-associated genes. With such models juxtaposed against tandem duplications, examples of Transposable Element (TE)-induced duplication are ripe for studies of genome evolution and effector duplication (Figure 3).

Applications for developing robust and durable SCN-resistant soybean cultivars may lie with understanding SCN effectors. To facilitate effector identification, we incorporated numerous effector-related analyses into SCNBase and created detailed category pages to host supporting information. A first step to delimiting the genes important for the host–parasite exchange lies with identifying secreted proteins. A ‘secreted’ JBrowse track displays this information, identifying 3341/29 769 genes encoding a 5′ secretion signaling peptide and lacking a transmembrane domain. Secretory proteins without transmembrane domains and expressed in SCN gland cells are deemed effectors and may be directly involved in parasitism. Our genome publication (31) predicted a total of 431 putative effector proteins using Gmap (48) alignments and motif predictions from published effector sequences, as well as genes with promoters enriched for DOG-boxes. A total of 575 putative effectors could be identified by including 151 horizontally transferred genes, as the development of plant parasitism coincides with massive horizontal gene transfer (31, 55, 56). Including the newly identified ortholog-based effector-like genes creates a set of 3324 putative effector genes; however, only 495 of these proteins are predicted to be secreted. These genes can be further distilled through comparisons with DNA transposons (65), LTR retrotransposons (23) and tandem duplications (173) to reveal duplicated and secreted effector genes that may be copy-number dependent. Thus, by combining the numerous data sets on SCNBase, scientists can create the crucial contrast needed to identify interesting gene targets.

### Conclusion and future perspective

SCN is considered the greatest threat to soybean yield worldwide but was lacking many genomic resources until now. SCNBase fills this void and will open avenues of research and increase the efficiency of many experimental approaches. To incorporate ever-increasing quantities of data, SCNBase is designed for continual improvement by encouraging extensive community-based curation through the use of standardized forms. Furthermore, an improved selection of bioinformatic tools will continue to be implemented, thereby increasing online accessibility and analyses.

## Notes

**Database URL**: https://scnbase.org/
